# Use of* Moringa oleifera* Flower Pod Extract as Natural Preservative and Development of SCAR Marker for Its DNA Based Identification

**DOI:** 10.1155/2016/7584318

**Published:** 2016-07-04

**Authors:** Iram Gull, Attia Javed, Muhammad Shahbaz Aslam, Roohi Mushtaq, Muhammad Amin Athar

**Affiliations:** ^1^Institute of Biochemistry and Biotechnology, University of the Punjab, Quaid-i-Azam Campus, Lahore 54590, Pakistan; ^2^Department of Biochemistry and Biotechnology, University of Sargodha, Lahore Campus, Lahore 53800, Pakistan

## Abstract

The use of* Moringa oleifera* as natural food preservative has been evaluated in the present study. In addition, for quality assurance, the study has also been focused on the shelf life of product to authenticate the identification of plant by development of DNA based marker. Among the different extracts prepared from flower pods of* Moringa oleifera*, methanol and aqueous extract exhibited high antibacterial and antioxidant activity, respectively. The high phenolic contents (53.5 ± 0.169 mg GAE/g) and flavonoid contents (10.9 ± 0.094 mg QE/g) were also recorded in methanol and aqueous extract, respectively. Due to instability of bioactive compounds in aqueous extract, methanol extract is considered as potent natural preservative. The shelf life of methanol extract was observed for two months at 4°C under dark conditions. The developed SCAR primers (MOF_217/317_/MOR_317_) specifically amplified a fragment of 317 bp from DNA of* Moringa oleifera* samples collected from different regions of Punjab province of Pakistan. The methanol extract of* Moringa oleifera* flower pods has great potential to be used as natural preservative and nutraceutical in food industry.

## 1. Introduction

Foodborne diseases are global issue with significant impact on human health [1]. According to the World Health Organization report (2015), ~1 in 10 people in the world, fall ill after consuming contaminated food annually [2]. Food protection from microbial and chemical deterioration is essential in food industry. Food borne pathogens such as* E. coli*,* S. aureus*,* S. typhi*,* Shigella*, and* P. aeruginosa* not only cause foodborne illnesses but also spoil food products [3]. Generally, chemically synthesized preservatives including butylated hydroxyl anisole (BHA), butylated hydroxyl toluene (BHT), tertiary butylated hydroquinone (TBHQ), and propyl gallate (PG) are used in food industry to avoid microbial and oxidative deterioration of food products [4]. However, serious concerns have been raised against the use of synthetic preservatives due to the health risk issue and toxicity [5].

With increase in consumer consciousness about safety of food additives, there is a growing need to search safer alternatives [6]. Plants are the richest biosource of natural compounds having antioxidant [7], antimicrobial [8], and antiviral activity [9]. Plant extracts are safe natural substitute to chemical food additives to avoid microbial and oxidative food spoilage. Before large scale production and use of plant extracts, it is important to consider the stability of plant extracts over the period of time under different storage conditions [10].

Additionally, authentic identification of therapeutically important plants to prevent advertent or inadvertent adulteration by low quality plants is a serious issue [11]. Substitution or adulteration of a medicinal plant with low quality inherent toxic herb by fraudulent actions can negatively affect the quality of medicinal plants [12]. Generally, for standardization and quality control, macroscopic, microscopic (morphology and histology), and chemical profiling (HPLC, TLC, and GC) of medicinal plants are in practice for their authentication [13–15]. Microscopic and macroscopic studies need to be conducted by expert personnel. Further, the chemical composition of plants is affected by change of growth stage, environmental factors, storage conditions, and harvesting process. The molecular authentication procedures are considered as more precise, specific, and suitable for medicinal plant identification in comparison to above discussed methods because DNA based techniques are independent of growth stage, environmental factors, and physical form of plant material [16, 17].

*Moringa oleifera* is a plant of family Moringaceae. Various parts of the plant are used as nutritious food commodity in many countries, that is, Pakistan, India, Philippines, and Hawaii and in some parts of Africa [18]. In underdeveloped countries (Senegal and Haiti), powder of leaves of* Moringa oleifera* has also been used to treat malnutrition in children, pregnant women, and nursing mothers [19]. The leaves of* Moringa* has more iron than spinach, more calcium than milk, more potassium than banana, and more vitamin C than oranges and the protein quality rivals the egg and milk protein [20]. For a long time, it has also been used as traditional medicine for treatment of many diseases [21–23].

Therefore, the objectives of the present study are (i) to evaluate the antimicrobial and antioxidant activity of* Moringa oleifera* flower pods and (ii) to develop SCAR marker using leaves as plant material for authentic identification of this medicinal plant.

## 2. Materials and Methods

### 2.1. Collection of Plant Material and Preparation of Crude Extracts

Fresh flower pods of* Moringa oleifera* were purchased from the local market of Lahore, Pakistan, in the month of March and April 2014, and authenticated morphologically from Department of Botany, University of the Punjab, Lahore, Pakistan. The flower pods (500 g) were completely air-dried and pulverized mechanically into powder using electric grinder. The powder (5 g) was extracted by soaking in 50 mL of each solvent (methanol, ethanol, water, chloroform, and acetone) separately in screw capped 250 mL Erlenmeyer flasks for 24 hours with shaking at 160 rpm in orbital shaker at ambient temperature. The extracts were filtered through eight layers of muslin cloth and then centrifuged at 8000 ×g for 15 minutes. The clear supernatant was collected and dried at 45°C in rotary evaporator (Laborota 4000-efficient, Heidolph). All the dried residues were dissolved in Dimethylsulfoxide (DMSO) except aqueous extract residues which were dissolved in water to known concentrations. The extracts were stored at 4°C till further use.

### 2.2. Bacterial Strains

Six bacterial strains (*E. coli*,* S. typhi*,* Shigella*,* S. aureus*, and* B. subtilis*) isolated from different food samples after biochemical characterizations [24] were used in the present study.

### 2.3. Antibacterial Activity Assay

The antibacterial activity assay was performed by disc diffusion method [25]. Sterile nutrient agar plates were seeded with 10^7^ CFU/mL of each bacterial strain under aseptic conditions. The sterile discs of Whatman Number 1 filter paper (5 mm) were dipped in extracts for 5 minutes and placed on the surface of seeded plates and pressed gently. The plates were incubated overnight at 37°C. The disc soaked in DMSO was used as negative control. The test was performed in triplicate for each bacterial strain. The antibacterial activity was recorded as mean of diameter of zone of inhibition (mm).

### 2.4. Minimum Inhibitory Concentration (MIC)

The minimum inhibitory concentration (MIC) was determined as described by Bag and Chattopadhyay [26]. MIC assay was performed in 96-well microtitre plate. Each well containing 90 *μ*L of nutrient broth was inoculated with 10 *μ*L of each bacterial culture (5 × 10^5^ CFU/mL). The extracts (100 *μ*L) with concentrations ranging from 900 mg/mL to 6.25 mg/mL were added in each well separately. After overnight incubation of plate at 37°C, 40 *μ*L of 0.4 mg/mL p-iodonitrotetrazolium violet (INT) was added in each well and incubated again for 6 hours. After incubation, the plates were observed for change in color from yellow to red to purple for viable bacteria. The lowest concentration of plant extract with no color change was recorded as MIC. The experiment was performed in triplicate.

### 2.5. Time-Kill Kinetic Analysis

The bacterial killing rate was determined by time-kill kinetic assay at 1x MIC as described in our earlier study [27]. The cultures collected at different time (0–24 hours) were serially diluted and used to determined viable cell count. The results were recorded as logarithm of CFU against incubation time.

### 2.6. Storage Stability Assay

The stability of* Moringa oleifera* flower pod extracts was evaluated over the period of time with and without their exposure to light while being stored at different temperatures (−20°C, 4°C, and room temperature). At the end of each storage time, the stability of antimicrobial compounds in crude extracts was determined by antimicrobial assay as described previously.

### 2.7. Antioxidant Assay

#### 2.7.1. Ferric Reducing Power Assay (FRPA)

Ferric reducing power assay was performed as described by Mohamed et al. [28]. Briefly, 100 *μ*L of each extract was mixed with 2.5 mL of 200 mM phosphate buffer pH 6.6 and 2.5 mL of 1% potassium ferricyanide [K_3_Fe(CN)_6_]. After incubation at 50°C for 20 minutes, 2.5 mL of 10% trichloroacetic acid was added to mixture and centrifuged at 10,000 rpm for 10 minutes. The upper layer of solution (5 mL) was mixed with 5 mL of distilled water and 1 mL of 0.1% ferric chloride. The absorbance was measured at 700 nm. Ascorbic acid was used as standard and results were expressed by plotting absorbance against concentration.

### 2.8. Phytochemical Studies

The extracts of* Moringa oleifera* were screened for the presences of major phytochemicals such as phenolics, flavonoids, alkaloids, terpenoids, tannins, steroids, glycosides, and anthraquinone using standard procedures as described in Trease and Evans [29] and Sofowora [30].

#### 2.8.1. Phenolic Compounds

To 1 mL of extract, add 4 drops of ethanol and 3 drops of 0.1% FeCl_3_. The appearance of red color indicated the presence of phenolic compounds.

#### 2.8.2. Anthraquinone

To 2 mL of plant extract, 1 mL of diluted 10% ammonia was added. The pink red coloration in ammoniacal (lower) layer showed the presence of anthraquinone.

#### 2.8.3. Flavonoids

1 mL of extract was diluted with water to 5 mL. Then, 2 mL of 10% NaOH was added to develop yellow color. The change from yellow color to colorless solution by the addition of diluted HCL indicated the presence of flavonoids.

#### 2.8.4. Alkaloids

1 mL of extract was diluted with 1% HCL up to 5 mL. To the 1 mL of the diluted extract, few drops of Dragendorff's reagent were added. The orange red precipitates indicated the presence of alkaloids.

#### 2.8.5. Terpenoids

To 1 mL of extract, 2 mL of chloroform was added. Then, 2 mL of H_2_SO_4_ was added. The reddish brown color at interface confirmed the presence of terpenoids in extract.

#### 2.8.6. Tannins

Few drops of 1% FeCl_3_ were added to the 1 mL of extract. The presence of brownish green precipitates indicated the presence of tannins.

#### 2.8.7. Glycoside

To 1 mL of extract, 2 mL of glacial acetic acid and a drop of 5% FeCl_3_ were added. The development of brown ring indicated the presence of glycoside.

#### 2.8.8. Steroids

To 1 mL of extract, few drops of concentrated H_2_SO_4_ were added. The appearance of red color indicated the presence of steroids.

### 2.9. Estimation of Total Phenolic Contents

**Figure 1:**
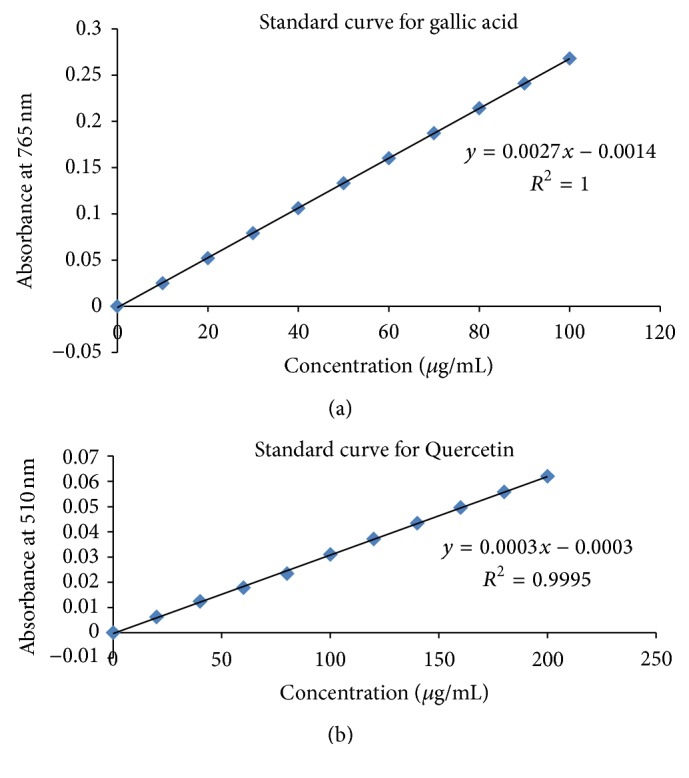
(a) Standard curve of gallic acid; (b) standard curve of Quercetin.

Total phenolic contents were estimated in extracts using Folin Ciocalteu reagent based assay as described by Barku et al. [31] with slight modifications. Briefly, 150 *μ*L of extract was mixed thoroughly with 750 *μ*L of Folin Ciocalteu reagent (diluted 10 times with distilled water) and then added 600 *μ*L of 75 g/L Na_2_CO_3_. The mixture was placed at room temperature for 30 minutes for color development. The absorbance of developed blue color was recorded at 765 nm. Gallic acid was used as standard for calibration curve (Figure 1(a)). The total phenolic contents were expressed as milligram (mg) of gallic acid equivalent (GAE)/gram (g) of dry weight.

### 2.10. Estimation of Total Flavonoids

Total flavonoid contents were estimated as described by Manian et al. [32]. Briefly, 150 *μ*L of each plant extract was diluted up to 1 mL with distilled water, followed by addition of 45 *μ*L of 5% sodium nitrite. After incubation for 5 minutes at room temperature, 65 *μ*L of 10% aluminium chloride was added and allowed to stand for 6 minutes at room temperature. After that, 300 *μ*L of 1 N NaOH was added and absorbance was taken at 510 nm. Quercetin was used as standard for calibration curve (Figure 1(b)). Total flavonoid contents were expressed as mg/g of Quercetin equivalent.

### 2.11. Statistical Analysis

Each experiment was performed in triplicate. The mean standard error and one way ANOVA were performed by SPSS version 21.0 software. The pairwise significant difference between the means of treatment levels was assessed by Duncan's multiple range test at *p* < 0.05.

### 2.12. SCAR Marker Development

Leaf samples of* Moringa oleifera* were collected from different localities of Punjab, Pakistan. Leaves were washed and stored at −80°C. DNA was isolated using method described by J. J. Doyle and J. L. Doyle [33]. Random Amplification of Polymorphic DNA (RAPD) PCR was performed to develop DNA finger prints using 17 random decamers (Table 1). The PCR reaction mixture (25 *μ*L) included 0.5 *μ*L of* Moringa oleifera* DNA (100 ng/*μ*L), 2.5 *μ*L of 10 *μ*M random decamer, 0.5 *μ*L of 10 mM dNTPs, 2 *μ*L of MgCl_2_, 2.5 *μ*L of Taq buffer, and 0.25 *μ*L of Taq DNA polymerase (5 u/*μ*L) and volume was made up to 25 *μ*L with sterilized water. The amplification reactions were performed by initial denaturation at 95°C for 1 minute followed by 30 cycles of denaturation at 95°C for 1 minute, annealing at 40°C for 45 seconds, and extension at 72°C for 1 minute. RAPD-PCR patterns were analyzed on 1% agarose gel electrophoresis. The monomorphic band in all samples was excised from gel, purified, ligated into pTZ57R/T vector, and finally used for transformation of* E. coli* DH5*α* competent cells. Recombinant clones were selected by blue white screening [34]. The plasmid was isolated from white colonies and subjected to digestion with* xbaI*. The linearized plasmid size was equal to the size of vector pTZ57R/T and the size of the insert which confirmed successful cloning of DNA fragment of interest. The cloned DNA fragment was sequenced commercially. The nucleotide sequence of DNA fragment was used to designed SCAR primer pair (MOF_271/371_ and MOR_271_, MOR_371_). The amplicon using MOF_271/371_ and MOR_371_ was used as template for PCR amplification using MOF_271/371_ and MOR_271_ SCAR primers. The SCAR primer pair was used to amplify* Moringa oleifera* DNA isolated from different localities of Punjab using same PCR conditions except 2.5 *μ*L of each 10 uM MOF_271/371_, MOR_271_, and MOR_371_ SCAR primer instead of random decamer in reaction mixture and annealing at 66°C. The amplification was analyzed by 1% agarose gel electrophoresis.

## 3. Results and Discussion

In food industry, plant antimicrobial compounds have great potential to be used as biopreservative. In addition, the antioxidant activity of plant extracts prevents the oxidative degradation of food stuff. Plants are the natural source of antimicrobial and antioxidant compounds. Use of plant extracts having both activities improves food quality and also increases the acceptability of plant extracts as a replacement to the synthetic preservatives [35].

### 3.1. Antibacterial Activity

The extracts of flower pods of* Moringa oleifera* prepared in solvents of different polarity were examined for antibacterial activity against food borne pathogens (*Bacillus subtilis*,* E. coli*,* S. aureus*,* Salmonella typhi*, and* Shigella*) using disc diffusion method. The extracts showed broad spectrum of activity against these pathogens. The results are shown in Table 2. All the extracts showed antimicrobial activity against the used bacterial strains. The highest antibacterial activity against all used strains was recorded with methanol extract, while the lowest was observed with aqueous extract. The descending order of antimicrobial activity of different extracts against tested bacterial strains was as follows: methanol extract > acetone extract > chloroform extract > ethanol extract > aqueous extract. The descending order of sensitivity of bacterial strains for a different extracts was as follows:* methanol extract: Shigella* >* E. coli* >* B. subtilis* >* S. typhi*;* ethanol extract: S. typhi* >* B. subtilis* >* E. coli* >* Shigella*;* chloroform extract: S. typhi* >* E. coli* >* Shigella* >* B. subtilis*;* aqueous extract: E. coli* >* B. subtilis* >* Shigella* and* S. typhi*. The negligible antibacterial activity of aqueous extract of* Moringa* is in agreement with the results of the study reported by [36]. It is also reported in many studies that different parts of* Moringa oleifera* have been used to investigate their antimicrobial activity. However, sensitivity of pathogens to* Moringa* extracts prepared in polar to nonpolar solvents is variable. Some pathogens are sensitive to ethyl acetate/acetone extracts prepared from root bark [37, 38], whereas other pathogens showed high sensitivity to methanol extract prepared from stem bark [39]. Chloroform extract from seeds and ethanol extract of* Moringa* leaves has also been reported as effective against pathogens [40]. In our study, methanol extract of* Moringa oleifera* flower pods is observed as most effective against all tested food borne pathogens. It is concluded that difference in sensitivity of extracts prepared from different parts of* Moringa* is directly related to the concentration of antibacterial compounds in different parts of the same plant. Furthermore, the growth stage of plant, environmental/geographical factors, storage conditions, and difference in procedures of extract preparation also influence the chemical profile of plant and the concentration of antimicrobial compounds in extracts of same part of same plant.

### 3.2. Minimum Inhibitory Concentration (MIC)

The results are presented in Table 3. The lowest MIC value was recorded with methanol extract against all investigated food pathogens. The lowest MIC value for* S. typhi* was 12.5 mg/mL, followed by 17.5 mg/mL for* E. coli* and 25 mg/mL for* B. subtilis* and* Shigella*. The high MIC values were observed for aqueous extract (916 mg/mL). The MIC values clearly indicated that, among different extract of flower pods of* Moringa oleifera*, methanol extract renders promising antibacterial activity against number of food borne and food spoiling bacteria. In the study of Bukar et al. [40],* Shigella* was resistant to ethanol and chloroform extract prepared from leaves and seeds of* Moringa oleifera*, while* E. coli* was found to be sensitive to ethanol extract at concentration of 200 mg/mL and 500 mg/mL, respectively. Despite following the same procedure to prepare the extracts, the variation in sensitivity of food pathogens can be noticed against extracts prepared from different parts of* Moringa oleifera*. It leads to a conclusion that sensitivity of food pathogens varies from extract to extract prepared from different parts of the same plant.

### 3.3. Time-Kill Kinetic Analysis

**Figure 2:**
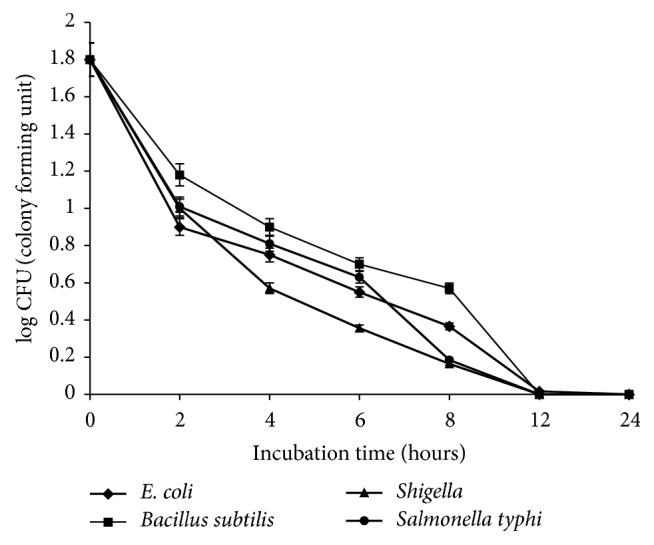
Time-kill kinetic assay for tested bacterial strains at 1x MIC. Values are mean of three independent experiments.

Time-kill kinetic assay was performed with methanol extract to determine the time duration during which the bacteria under investigation are killed. The results are shown in Figure 2. It was observed that the viable count of bacteria was significantly decreased after each specified interval of time. After 2 hours, 50% decrease in log CFU was recorded for* E. coli*, 34% for* B. subtilis*, and 44% for* S. typhi* and* Shigella*. After 12 hours, decrease in log CFU was achieved up to 99% for* E. coli*, 75% for* B. subtilis*, and 95% for* S. typhi*, and* Shigella* after 12 hours. No viable count was recorded after 24 hours. It was depicted from results that antimicrobial effect of extracts is increasing proportionately to time duration against the pathogen under investigation. The significant reduction (3 log_10_ CFU) was observed between 6 and 8 hours of bacterial exposure to extract.

### 3.4. Phytochemical Analysis

The plant extracts with known antimicrobial activity are of great importance in food preservation. The bioactive chemical substances (polyphenols, flavonoids, alkaloids, terpenoids, tannins, and others) are mainly responsible for rendering a definite action on microbial and chemical quality of food [41]. The phytochemicals analysis of different extracts of* Moringa oleifera* was also conducted (Table 4). The preliminary phytochemical analysis showed that flavonoids, phenol, tannins, and glycosides were present in all extracts which indicated that these are the major secondary metabolites in flower pods of* Moringa oleifera*. However, quantity of compounds is commensurate with the polarity of solvent and the part of plant under study [42]. The variation in composition of secondary metabolites in different parts (leaves, seeds, and bark) of* Moringa oleifera* has also been reported [43–46]. In present study, methanol extract contained all the tested phytochemicals except anthraquinone. The anthraquinone was not detected in any of the extracts of* Moringa oleifera* flower pods. In aqueous and chloroform extracts, all tested phytochemicals were present except steroids and anthraquinone. In ethanol and acetone extract, alkaloids and terpenoids were also not detected. The results of antibacterial assay of different extracts and their phytochemical analysis showed that combination of different compounds may be responsible for bioactivity of plant extracts. The diversity of phytochemicals in plant extracts provides good opportunity to effectively control the microbial growth as some microorganisms are not killed by single/pure antimicrobial compound [47].

#### 3.4.1. Total Phenolic Contents (TPC)

The total phenolic contents of* Moringa oleifera* flower pods are presented in Table 5. The results revealed significant variation in total phenolic contents in different extracts. The highest TPC was present in methanol extract (53.8 ± 0.169 mg GAE/g), while lowest in chloroform extract (14.1 mg GAE/g). The results of our study corroborate the results of other studies discussed below. Sohaimy et al. [48] showed that among the different extracts of the* Moringa oleifera* leaves, methanol extract had the highest amount of TPC (48.35 mg GAE/g). Similarly, Abdulkadir et al. [49] also reported high TPC (48.04 mg GAE/g) in methanol extract. Alhakmani et al. [50] reported low TPC (19.31 mg GAE/g) in ethanol extract of* Moringa oleifera* flower pods. The comparable results were also recorded in the present study as low TPC (25.93 mg GAE/g) in ethanol extract was noticed compared to methanol extract (48.35 mg GAE/g). The slight difference in TPC might be due to the difference in geographical origin of plant. Both the high TPC and high antimicrobial activity of methanol extract represent that phenolic compounds may be responsible for antimicrobial activity of methanol extract. It is pertinent to mention that methanol extracts contained most of the secondary metabolites from plants [28]. Therefore, the bioactivity of methanol extracts can be explained in terms of high TPC. The aqueous extract contained high TPC but showed negligible antibacterial activity that might be due to instability of active compounds in water.

#### 3.4.2. Total Flavonoid Contents (TFC)

The flavonoids constitute major component of plant phenolic compound and exert positive effect on human health. The flavonoid contents in different extracts prepared from flower pods of* Moringa oleifera* are summarized in Table 5. Total flavonoid contents ranged from 10.9 ± 0.094 mg QE/g to the 3.41 ± 0.006 mg QE/g. The highest contents were recorded in aqueous extract (10.9 ± 0.094 mg QE/g). Hence, water was most effective in extraction of flavonoids from flower pods of* Moringa*. Akhtar et al. [51] reported 6.9 ± 2.0 mg QE/g in methanol extract and 9.1 ± 3.5 mg QE/g in aqueous extract prepared from leaves and bark of* Moringa oleifera* which are lower than the flavonoid contents of methanol extract (9.13 mg QE/g) and aqueous extract (10.9 mg QE/g) prepared from flower pods that were recorded in the present study. Therefore, for acquisition of high flavonoid contents, aqueous extract of* Moringa oleifera* flower pods is potent natural biosource.

### 3.5. Ferricyanide Reducing Power Assay (FRPA)

Antioxidant activity of plant extracts prevents the cell and tissue damage caused by reactive oxygen species (ROS). Reducing power of plant extract indicates its antioxidant activity [52]. The bioactive compounds in plant extracts convert the ROS into more stable products by donation of electrons [53].

**Figure 3:**
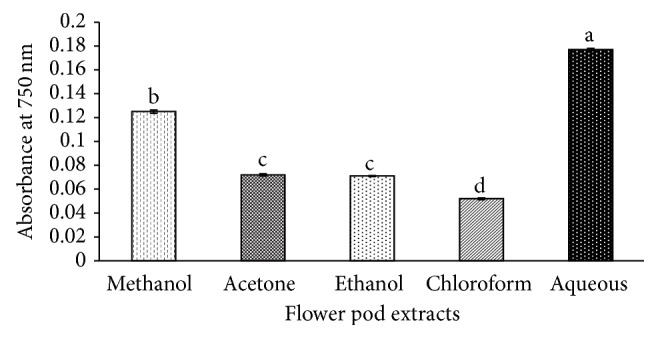
Antioxidant activity of different extracts of* Moringa oleifera* flower pods by Ferricyanide Reducing Power Assay (FRPA). Mean values having the same letter do not differ significantly (*p* < 0.05) according to Duncan's multiple range test.

In the present study, the reducing power of* Moringa oleifera* flower pod extracts was investigated by reducing Fe^+3^ ions to Fe^+2^ ions. The results displayed in Figure 3 showed that the aqueous extract had the highest reducing power followed by methanol extract. The chloroform extract showed the lowest reducing power. The high reducing power of methanol and aqueous extract can be correlated to the high phenolic and flavonoid contents estimated in these extracts. Phenolic compounds are considered as effective hydrogen donor that makes them good antioxidant [54]. The results of the present study corroborate the results of previous studies where it has been reported that plant phenolic compounds govern antioxidant activity of plant extracts [28, 55–57]. Therefore, aqueous extract of* Moringa oleifera* flower pods can be considered as a potent source of natural antioxidants.

High flavonoids and TPC in aqueous and methanol extract might be responsible for its high bioactivity (antibacterial and antioxidant activity). However, stability of compounds in methanol extract makes it appropriate choice as natural preservative with promising antibacterial and antioxidant activity.

### 3.6. Storage Stability of* Moringa oleifera* Extracts

**Figure 4:**
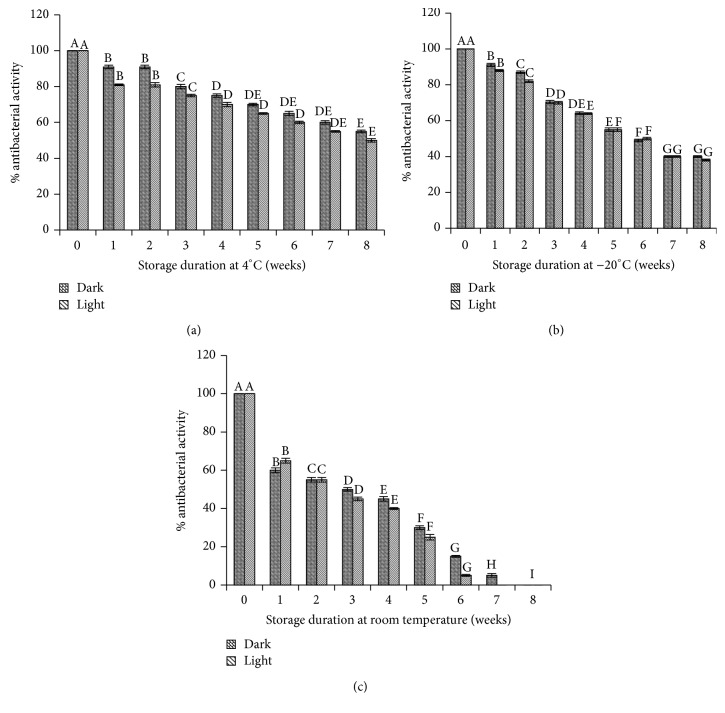
Stability of methanol extract at different storage temperatures for the period of two months with and without exposure of light. (a) Stability of extract at 4°C; (b) at −20°C; (c) at room temperature. Mean values having the same letter do not differ significantly (*p* < 0.05) according to Duncan's multiple range test.

Taking into consideration the pharmaceutical and nutraceutical importance of herbal extracts, it is also important to validate their stability and functionality over the period of time. It has been reported that functional products in plant extracts are sensitive to many factors, that is, light, storage temperature, and storage duration [10]. In the present study, stability of* Moringa oleifera* extracts was determined to ensure the stability and functionality of extract over the period of time. The extract was stored at three different temperatures (−20°C, 4°C, and room temperature) with and without exposure to light for the period of two months. The results shown in Figure 4 indicated that ~5% decrease in bioactivity of extracts was observed with extracts exposed to light compared to extracts stored under dark conditions. Decrease in bioactivity of extract was observed over the period of time. Further, rapid and significant decrease in bioactivity of extract was recorded during storage at room temperature (Figure 4(c)). After storage of one week at room temperature, 40% decrease in bioactivity was observed and this decrease reached to 60% after four weeks. The total bioactivity was lost after storage of six weeks at room temperature. At low temperatures, the extracts were quite stable. At 4°C, extract maintained its activity up to 75% after one month storage and showed 70% activity even after two months (Figure 4(a)). The extract lost its bioactivity up to 60% after storage of two months at −20°C (Figure 4(b)). Therefore, it is recommended to store the* Moringa oleifera* extracts at 4°C in brown bottles. The shelf life of the extract is 2 months at this storage temperature.

### 3.7. SCAR Marker for* Moringa oleifera*

**Figure 5:**
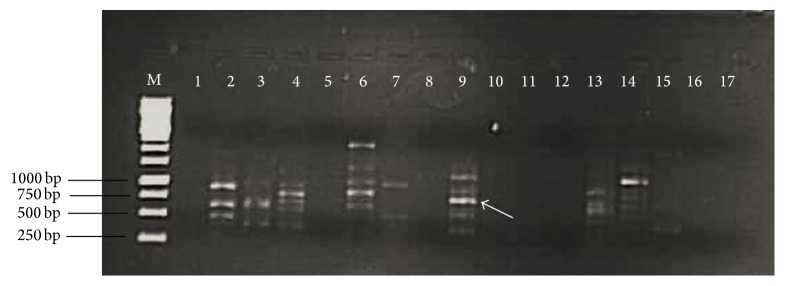
1% agarose gel electrophoretic analysis of RAPD pattern of* Moringa oleifera* DNA using 17 random decamers OPA 1–OPA 17. Lane M, DNA size marker; Lane 1–17, RAPD pattern of* Moringa oleifera* using OPA 1–OPA 17. OPA 9 gave reproducible same banding pattern with DNA sample of* Moringa oleifera* collected from different localities of Punjab province. Arrow indicates the selected monomorphic fragment (750 bp) for cloning and sequencing.

**Figure 6:**
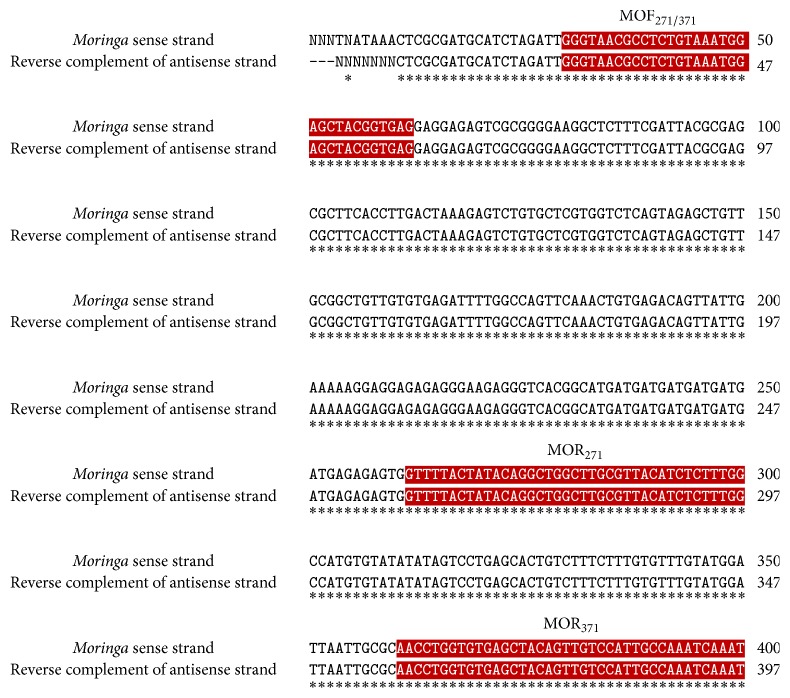
Comparison of sense strand and reverse complement of antisense strand sequence of selected fragment of* Moringa oleifera* from RAPD pattern of OPA 9 using ClustalW program. MOF_271/371_, highlighted by red color is indicating the region selected for forward primer. MOR_271_ and MOR_371_ highlighted by red color are indicating the region selected for designing reverse primer. The symbol *∗* indicates the homology between the sequences.

**Figure 7:**
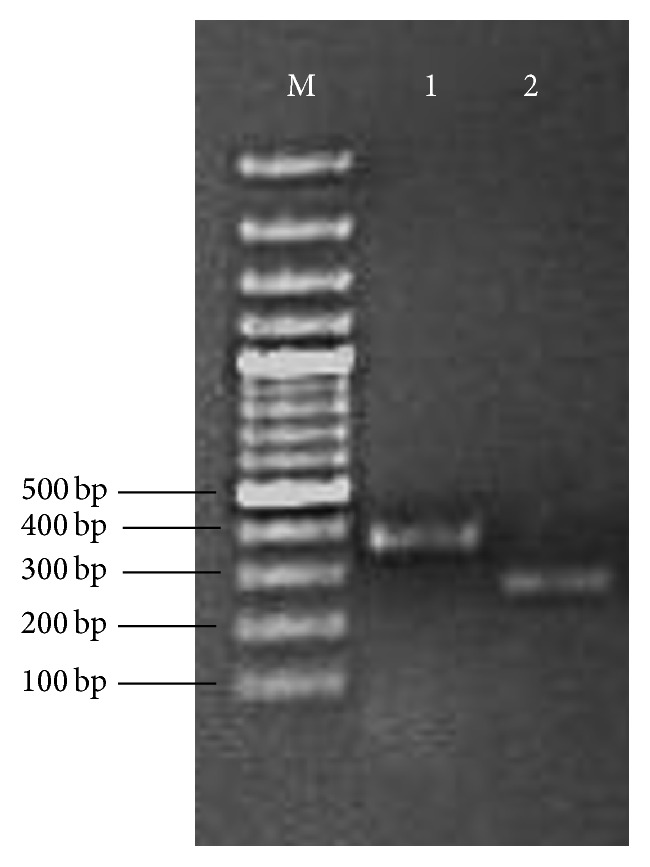
1% agarose electrophoresis analysis of PCR products amplified with developed SCAR primers for* Moringa oleifera*.* Lane M*: DNA size marker;* Lane 1*: PCR product of 317 bp using SCAR primer MOF_217/317_/MOR_317_;* Lane 2*: PCR product of 217 bp using 317 bp amplicon as template with MOF_217/317_ and MOR_217_ SCAR primers.

With revival of herbal medicine in modern world, it has utterly become indispensable to deal with the issues related to the quality, purity, authentication, and standardization of raw plant material.* Moringa oleifera* has great potential as a natural substitute to chemical food preservatives and as a natural antioxidant. In addition to this, authentic identification of this valuable plant is also highly crucial. Generally used organoleptic, anatomical, and analytical techniques have several drawbacks and limitations such as being time-consuming, being expensive, being affected by environmental factors, and requirement of botanical experts [58, 59]. Keeping in view the limitations and drawbacks of these techniques, the present study focused on molecular authentication of* Moringa oleifera* by converting RAPD (Random Amplification of Polymorphic DNA) marker into SCAR (Sequence Characterized Amplified Region) marker. The DNA isolated from* Moringa oleifera* leaves samples collected from different regions of Punjab, Pakistan, was amplified to develop RAPD pattern. OPA 9 primer showed reproducible banding pattern (Figure 5). The fragment of ~750 bp (indicated by arrow) monomorphic in all samples was excised from gel, purified, and cloned. The positive transformants were sequenced using universal M13 primers. To ensure the accuracy of sequence, both (sense and antisense) strands of fragment were sequenced. The reverse complement sequence of antisense strand was compared with sequence of sense strand using freely available online program ClustalW. The sequence is shown in Figure 6. The sequence was submitted to Genbank and Accession number is KU247867. The sequence was used to design SCAR primers (MOF_271/317_, MOR_217_, and MOR_317_). The primers were synthesized commercially. The expected size of amplicon using MOF_271/317_ and MOR_317_ was 317 bp. To increase the precision of developed SCAR markers, the 317 bp amplicon was used as template for primer pair MOF_271/317_ and MOR_217_. The expected size of amplicon was 217 bp. The developed SCAR primers (Table 6) were used to amplify DNA from* Moringa oleifera* samples randomly collected from different localities of Punjab province. A fragment of 317 bp was successfully amplified from all samples using developed MOF_217/317_  and MOR_317_ SCAR primers (Figure 7). In second round of PCR, using 317 bp amplicon as template with MOF_217/317_  and MOR_217_ also successfully amplified a fragment of 217 bp in all samples (Figure 7). The conversion of RAPD marker into SCAR marker simplifies the identification procedure. Instead of complex banding pattern and lack of reproducibility of RAPD, SCAR marker relies on amplification of a single band [60]. Two rounds of PCR increase the accuracy and reliability of developed SCAR markers. SCAR marker based identification is more simple and straightforward than other molecular techniques, that is, intersimple sequence repeat (ISSR), Amplified Fragment Length Polymorphisms (AFLP), Simple Sequence Repeats (SSR), and RAPD. In a simple PCR reaction, using long primers, a specific band can be amplified. The SCAR marker developed in the present study simplifies the identification of* Moringa oleifera* samples collected from of Punjab, Pakistan, just by a PCR reaction.

*Moringa oleifera* has gained widespread popularity due to potent antibacterial [37, 61, 62], antifungal [63, 64], antioxidant [65], antiproliferative [66] antidiabetic [67], anti-inflammatory [68], cholesterol lowering [69], and hepatoprotective [70] activities of its extracts prepared from different parts (root, bark, stem, leaves, seeds, fruit, and flower). These phytochemicals have been reported to be involved treatment of diseases. The phenols and flavonoids act as antioxidants that prevent oxidative damage and control degenerative diseases [71]. Alkaloids are known for antibacterial and antifungal activities [72], whereas glycosides have been reported for regulation of heart beat and treatment of congestive heart failure [73]. Tannins have been reported for treatment of diabetes [74]. The methanol extract of* Moringa oleifera* flower pods contains number of phytochemicals (phenolic compounds, flavonoids, alkaloid, terpenoids, and tannins) and high total phenolic and flavonoid contents as recorded in the present study. These findings show the potential of methanol extract of* Moringa oleifera* flower pods for its use as natural preservative and nutraceutical in food industry.

## 4. Conclusion

The methanol extract of* Moringa oleifera* flower pods has vast potential as a nutraceutical and a natural substitute to synthetic food preservatives. Further research is necessary for real application of these extracts in food as extrapolation of results from* in vitro* studies to food products is not straightforward due to complex nature of food and different interconnecting environments. The identification at molecular level with the developed SCAR marker of* Moringa oleifera* is useful for authentication and quality assurance of this miracle plant.

## Figures and Tables

**Table 1:** Random decamers used for RAPD fingerprints of *Moringa oleifera*.

OPA-01	5′-CAG GCC CTT C-3′
OPA-02	5′-TGC CGA GCT G-3′
OPA-03	5′-AGT CAG CCA C-3′
OPA-04	5′-AAT CGG GCT G-3′
OPA-05	5′-AGG GGT CTT G-3′
OPA-06	5′-GGT CCC TGA C-3′
OPA-07	5′-GAA ACG GGTG-3′
OPA-08	5′-GTG ACG TAG G-3′
OPA-09	5′-GGG TAA CGC C-3′
OPA-10	5′-GTG ATC GCA G-3′
OPA-11	5′-CAA TCG CCG T-3′
OPA-12	5′-TCG GCG ATA G-3′
OPA-13	5′-CAG CAC CCA C-3′
OPA-14	5′-TCT GTG CTG G-3′
OPA-15	5′-TTC CGA ACC C-3′
OPA-16	5′-AGC CAG CGA A-3′
OPA-17	5′-GAC CGC TTG T-3′

**Table 2:** Antibacterial activity of flower pods extracts *Moringa oleifera*.

	Zone of inhibition in (mm)				
	Methanol extract (220 mg/mL)	Acetone extract (200 mg/mL)	Chloroform extract (430 mg/mL)	Ethanol extract (959 mg/mL)	Aqueous extract (916 mg/mL)
*Shigella*	19.3 ± 0.981^a^	14 ± 1.247^b^	12.6 ± 0.544^bc^	11 ± 0.471^bc^	10 ± 0.471^c^
*E. coli*	18.3 ± 1.186^a^	15.3 ± 0.720^b^	13.3 ± 1.186^bc^	11.6 ± 0.981^bc^	10.3 ± 0.54^c^
*B. subtilis*	17 ± 0.471^a^	13 ± 0.942^b^	13 ± 0.942^b^	10.6 ± 0.720^b^	10.3 ± 0.720^b^
*S. typhi*	17 ± 0.942^a^	14 ± 0.720^ab^	14 ± 1.247^ab^	13 ± 0.942^bc^	10 ± 0.471^c^

Values are mean of three independent experiments. Mean values followed by the different letters in the same row differ significantly (*p* < 0.05) according to Duncan's multiple range test.

**Table 3:** Minimum inhibitory concentration (MIC) of flower pods extracts of *Moringa oleifera*.

	Minimum inhibitory concentration (mg/mL)				
	Methanol extract	Acetone extract	Chloroform extract	Ethanol extract	Aqueous extract
*Shigella*	25 ± 0.00	27.5 ± 0.00	53.1 ± 0.00	119 ± 0.00	916 ± 0.00
*E. coli*	17.5 ± 0.00	25 ± 0.00	53.1 ± 0.00	119 ± 0.00	916 ± 0.00
*B. subtilis*	25 ± 0.00	27.5 ± 0.00	26.8 ± 0.00	119 ± 0.00	916 ± 0.00
*S. typhi*	12.5 ± 0.00	17.5 ± 0.00	107 ± 0.00	119 ± 0.00	916 ± 0.00

Values are mean of three independent experiments.

**Table 4:** Phytochemical analysis of flower pods extracts of *Moringa oleifera.*

		Methanol extract	Ethanol extract	Chloroform extract	Acetone extract	Aqueous extract
Alkaloid	Wagner's test	_+_	−	+	−	+
Anthraquinone	Borntrager's test	−	−	−	−	−
Flavonoid	Alkaline reagent test	+	+	+	+	+
Terpenoids	Salkowski test (modified)	+	−	+	−	+
Tannins	Braemer's test	+	+	+	+	+
Glycoside	Keller Kiliani test	+	+	+	+	+
Phenols	FeCl_3_ test	+	+	+	+	+
Steroids	Salkowski test	+	+	−	−	−

The sign “+” indicates “detection” and the sign “−” indicates “no detection” of compound in the extracts.

**Table 5:** Total phenolic and flavonoid contents in flower pod extract of *Moringa oleifera*.

	Total phenolic contents (mg GAE/g)	Total flavonoids (mg QE/g)
Methanol extract	53.8 ± 0.169^a^	9.13 ± 0.021^b^
Ethanol extract	25.93 ± 0.151^c^	8.70 ± 0.085^c^
Chloroform extract	14.1 ± 0.945^d^	3.41 ± 0.006^e^
Acetone extract	27.7 ± 0.124^c^	4.05 ± 0.026^d^
Aqueous extract	42.4 ± 0.094^b^	10.9 ± 0.094^a^

Values are mean of three independent experiments. Mean values followed by different letters in same column differ significantly (*p* < 0.05) according to Duncan's multiple range test.

**Table 6:** SCAR primers for *Moringa oleifera*.

MOF_217/317_	5′GGGTACGCCTCTGTAAATGGAGCTACGGTGAG 3′
MOR_217_	5′ATTTGATTTGGCAATGGACAACTGTAGCTCACACCAG GTT 3′
MOR_317_	5′CCAAAGAGATGTAACGCAAGCCAGCCTGTATAGTAAAAC 3′
